# Venous Thrombosis in Children with Acute Lymphoblastic Leukemia Treated on DCOG ALL-9 and ALL-10 Protocols: The Effect of Fresh Frozen Plasma

**DOI:** 10.1055/s-0039-1688412

**Published:** 2019-04-24

**Authors:** Irene L. M. Klaassen, Charlotte C. M. Zuurbier, Barbara A. Hutten, Cor van den Bos, A. Y. Netteke Schouten, Eva Stokhuijzen, C. Heleen van Ommen

**Affiliations:** 1 Department of Pediatric Hematology, Emma Children's Hospital, Amsterdam University Medical Centers, Amsterdam, The Netherlands; 2 Department of Vascular Medicine, Amsterdam University Medical Centers, Amsterdam, The Netherlands; 3 Department of Clinical Epidemiology, Biostatistics and Bioinformatics, Amsterdam University Medical Centers, Amsterdam, The Netherlands; 4 Department of Pediatric Oncology, Emma Children's Hospital, Amsterdam University Medical Centers, Amsterdam, The Netherlands; 5 Princess Máxima Center for Pediatric Oncology, Utrecht, The Netherlands; 6 Department of Pediatric Hematology, Erasmus Medical Center/Sophia Children's Hospital, Rotterdam, The Netherlands

**Keywords:** Acute lymphoblastic leukemia, fresh frozen plasma, pediatric, risk factors, venous thromboembolism

## Abstract

**Background** Venous thromboembolism (VTE) is an important complication for treatment of acute lymphoblastic leukemia (ALL) in children. Especially, ALL treatment, with therapeutics such as asparaginase and steroids, increases the thrombotic risk by reduction in procoagulant and anticoagulant proteins. Replacement of deficient natural anticoagulants by administration of fresh frozen plasma (FFP) may have a preventive effect on the occurrence of VTE.

**Methods** We retrospectively analyzed all consecutive children (≤18 years) with ALL, treated on the Dutch Childhood Oncology Group (DCOG) ALL-9 and ALL-10 protocols at the Emma Children's Hospital Academic Medical Center between February 1997 and January 2012, to study the effect of FFP on VTE incidence, antithrombin and fibrinogen plasma levels, and VTE risk factors.

**Results** In total, 18/205 patients developed VTE (8.8%; 95% confidence interval [CI]: 4.9–12.7%). In all patients, VTE occurred after asparaginase administration. In total, 82/205 patients (40%) received FFP. FFP supplementation did not prevent VTE or alter plasma levels of antithrombin or fibrinogen. In the multivariate analysis, VTE occurred significantly more frequently in children ≥12 years (odds ratio [OR]: 3.89; 95% CI: 1.29–11.73) and treated according to the ALL-10 protocol (OR: 3.71; 95% CI: 1.13–12.17).

**Conclusion** FFP supplementation does not seem to be beneficial in the prevention of VTE in pediatric ALL patients. In addition, age ≥12 years and treatment according to the DCOG ALL-10 protocol with intensive and prolonged administration of asparaginase in combination with prednisone are risk factors. There is a need for effective preventive strategies in ALL patients at high risk for VTE.

## Introduction

Venous thromboembolism (VTE) is an important complication for treatment of acute lymphoblastic leukemia (ALL) in children. The prevalence of VTE in pediatric ALL patients varies widely from 1.2 to 37% due to definition of thrombosis (asymptomatic or symptomatic), study design (retrospective or prospective), and treatment protocols. 1 The increased risk of VTE can be explained by different mechanisms. First of all, coagulation can be activated due to procoagulant substances, such as thrombin, or by impairment of fibrinolytic or anticoagulation pathways, or by the disease itself. Furthermore, treatment of ALL enhances the thrombotic risk. 2 Most thrombotic events occur during the administration of asparaginase and steroid therapy. Both drugs are vital components of ALL treatment protocols. Asparaginase catalyzes the hydrolysis of asparagine to aspartic acid and ammonia. As a consequence, the total amount of asparagine is decreased leading to reduction of protein synthesis, causing cell dead of the lymphoblasts. Due to reduced protein synthesis, both procoagulant and anticoagulant proteins are reduced, especially antithrombin (AT). 3 In addition, asparaginase seems to cause thrombin initiation by upregulating tissue factor as a result of activation of white cells and endothelium. Steroids add to the prothrombotic state, by increasing the von Willebrand/factor VIII complex and by inducing a hypofibrinolytic state. 4 Finally, additional factors such as infection and the use of central venous catheters (CVCs) contribute to the thrombotic risk. 2 5

As the survival rate of ALL patients has increased tremendously in the last few decades, it becomes ever more important to prevent mortality and morbidity of treatment-associated complications, such as VTE. 6 7 Mortality is reported to vary between 0 and 50%, with an average of 15%. 1 As half of the thrombi in ALL patients are located in the cerebral sinuses, neurologic sequelae may occur including epilepsy, neurological deficits, and chronic headaches. These complications are reported to occur in approximately 15 to 20% of the patients. 8 9 10 Furthermore, VTE may lead to catheter-related infection and occlusion, postthrombotic symptoms, and may have impact on the outcome of cancer as a result of delay or decrease of asparaginase therapy. 5 11

The high VTE risk in conjunction with these significant complications may warrant primary thromboprophylactic measures. These include replacement of deficient natural anticoagulants by the administration of fresh frozen plasma (FFP) or AT concentrate as well as the administration of anticoagulants, such as low-molecular-weight heparin (LMWH) or direct oral anticoagulants (DOACs). Until now, there is insufficient evidence to justify their use in pediatric patients with ALL. In 2013, however, a retrospective observational study in adults suggested a preventive effect of FFP supplementation on the occurrence of VTE incidence during ALL treatment in adults. 12 Therefore, we retrospectively studied the effect of FFP on VTE incidence and on the plasma levels of AT and fibrinogen in children with newly diagnosed ALL treated according to the Dutch Childhood Oncology Group (DCOG) ALL-9 and ALL-10 protocols during asparaginase therapy in one tertiary care center. Furthermore, the total incidence of VTE during the whole ALL treatment period and potential risk factors for development of VTE were assessed.

## Patients and Methods

### Study Design

This study is a retrospective observational study, which was performed in the Emma Children's Hospital (EKZ)/Academic Medical Center (AMC) in Amsterdam, the Netherlands between February 1997 and January 2012. To estimate the effect of FFP supplementation on fibrinogen and AT levels, a sub-analysis was done in patients with FFP supplementation.

### Patient Population

The patient population consisted of all consecutive children (≤18 years of age) with malignancies who received medical treatment according to the DCOG ALL-9 or ALL-10 protocols. Patients were excluded when they did not receive therapy according to the DCOG ALL-9 or ALL-10 protocols or data were unavailable. No approval of the medical ethics committee was required, as this was a retrospective study and participation in this study did not involve any deviation from normal clinical practice.

### DCOG ALL Treatment Protocols

In the Netherlands, pediatric ALL patients were treated according to the DCOG ALL-9 protocol from January 1997 until October 2004. From October 2004 until January 2012, they were treated according to the DCOG ALL-10 protocol. ALL was diagnosed according to standard criteria. In the DCOG ALL-9 protocol, patients were stratified into two risk groups: high risk group (HR) and non-high risk group (NHR). 13 In the DCOG ALL-10 protocol, minimal residual disease was an important tool to stratify into three risk groups: standard risk (SR), medium risk (MR), and high risk (HR). 14

**Fig. 1:**
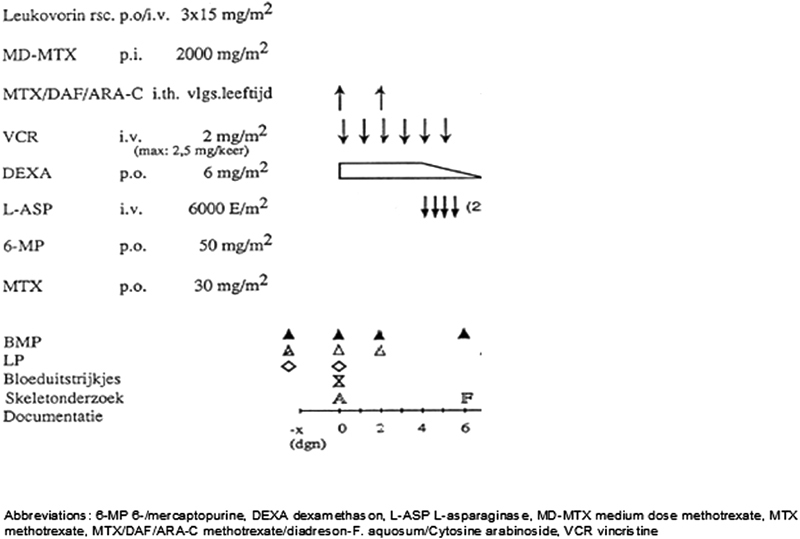
DCOG ALL-9 induction protocol. ALL, acute lymphoblastic leukemia; DCOG, Dutch Childhood Oncology Group.

**Fig. 2:**
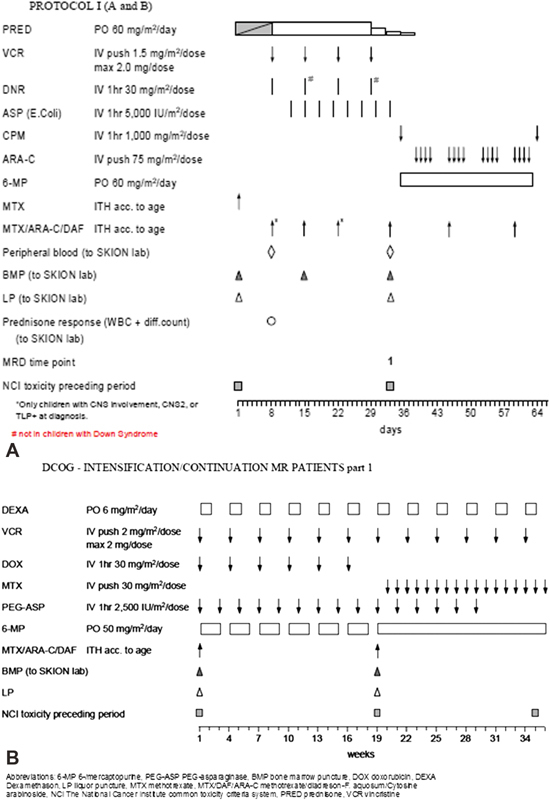
( **A** ) DCOG ALL-10 induction protocol 1A and B. ( **B** ) DCOG intensification/continuation MR patients part I. ALL, acute lymphoblastic leukemia; DCOG, Dutch Childhood Oncology Group, MR, medium risk.

The induction phases of ALL-9 and ALL-10 are shown in Fig. 1 and Fig. 2 , respectively. Treatment during the induction phase of the DCOG ALL-9 protocol included dexamethasone and four doses of 6,000 international unit (IU)/m ^2^ asparaginase intravenously. Treatment during the induction phase of the DCOG ALL-10 protocol included prednisone and eight doses of 5,000 IU/m ^2^ asparaginase intravenously. In the DCOG ALL-10 protocol, MR patients received intensification therapy containing dexamethasone (6 mg/m ^2^ /day for 5 days every 3 weeks) and Erwinia asparaginase (20,000 IU/m ^2^ per dose) three times per week during 30 weeks of intensification.

FFP supplementation was recommended if fibrinogen levels were below 1.0 g/L in the DCOG ALL-9 protocol and below 0.6 g/L in the DCOG ALL-10 protocol during asparaginase treatment . Plasma levels of fibrinogen were monitored before each asparaginase treatment day. Children received FFP supplementation by infusion of 10 mL/kg before administration of asparaginase, if there was a bleeding tendency and/or fibrinogen levels below 1.0 (DCOG ALL-9) or 0.6 g/L (DCOG ALL-10) on discretion of the treating physician. All patients received tunneled CVCs: Broviac or PAC. All patients received standard antibiotic and supportive care. Platelet transfusions were administered if platelet count were below 10 × 10 ^9^ /L in the induction phase.

### Data

Data were collected from the medical records of the included patients in the EKZ/AMC. Collected data included age at diagnosis, gender, details of ALL diagnosis (ALL immunophenotype and ALL risk group), body mass index, laboratory results, use of CVC, type of CVC, frequency of asparaginase administration, type of corticosteroid and use of thromboprophylactic measures (FFP, AT concentrate, unfractionated heparin, or LMWH), risk factors for VTE, the occurrence of VTE during induction phase and in subsequent treatment phases, and complications. AT and fibrinogen plasma levels were measured at diagnosis and before every administration of asparaginase in the induction phase and in the intensification phase during MR treatment.

Risk factors for VTE such as personal and family history of thrombosis, the presence of cardiac diseases, infection during asparaginase therapy, tumor lysis syndrome, or use of contraceptive drugs were recorded for each patient. From patients with VTE, additional data about clinical presentation and management were collected.

### Study Endpoints

The primary endpoints of this study were the overall incidence of symptomatic VTE during the whole ALL treatment period, and the incidence of symptomatic VTE during ALL asparaginase treatment with and without FFP. VTE was defined as an objectively diagnosed thrombosis on ultrasonography or neuroimaging (computed tomography [CT] or magnetic resonance imaging [MRI]). Secondary endpoints of this study were the risk factors for VTE and the plasma levels of AT and fibrinogen with and without FFP supplementation.

VTE was defined as asparaginase-related if VTE occurred within 21 days after asparaginase administration. Infection was defined as the development of pneumonia or sepsis during the induction phase. Pancreatitis was defined as a toxic effect as a result of ALL treatment. Tumor lysis syndrome was defined as the presence of two or more of the following metabolic abnormalities: hyperuricemia, hyperkalemia, and hyperphosphatemia. Fibrinogen and AT levels between 0.7 and 4 g/L and 80 and 140%, respectively, were regarded as normal.

### Statistical Analysis

Statistical analysis was conducted using SPSS software version 20.0.1 for Windows. The demographic data were analyzed using the chi-square test and the independent-samples *t* -test. The incidences of VTE occurrence were compared using the Fisher exact test. The cumulative VTE-free survival rate is calculated and shown with a Kaplan–Meier curve. The difference in VTE-free survival rates between patients treated according to DCOG ALL-9 and ALL-10 protocols were assessed using the log-rank test. A two-sided *p* -value of <0.05 was considered statistically significant. The risk factors were analyzed using logistic regression. Univariate and multivariate analyses with stepwise elimination were performed. All factors with statistically significant associations ( *p* < 0.05) for VTE were included in the multiple binary logistic regression model.

## Results

### Patients

Between the February 1, 1997 and January 2012, 286 patients were newly diagnosed with ALL in the EKZ/AMC and eligible for this study. Two hundred and five children treated according to the two DCOG ALL protocols were included. Eighty-one patients were excluded: 53 patients were mainly treated outside the EKZ/AMC after diagnosis, 15 patients were treated according to a different protocol, and 13 medical records were not available. Characteristics of the 205 patients who were enrolled in this study are shown in Table 1 .

**Table 1:** Demographic data of DCOG ALL-9 and ALL-10 protocol

Demographics	ALL-9 protocol	ALL-10 protocol	*p* -Value
Patients ( *n* , %)	113 (55.1%)	92 (44.9%)	
Age group			0.000
< 12 y	107 (95%)	72 (78%)	
≥ 12 y	6 (5%)	20 (22%)	
Median age in years (min–max)	5.2 (1–13.9)	7.3 (1.3–18)	0.000
Male ( *n* , %)	62 (54.8%)	61 (66.3%)	0.096
Type ALL:			0.000
B cell ( *n* , %)	112 (99.1%)	79 (85,9%)	
T cell ( *n* , %)	1 (0.88%)	13 (14.1%)	
CVC present (n, %)	96 (81.4%)	92 (100%)	0.000
FFP administration (n, %)	26 (23%)	56 (60.7%)	0.000

Abbreviations: ALL, acute lymphoblastic leukemia; CVC, central venous catheter; DCOG, Dutch Childhood Oncology Group; FFP, fresh frozen plasma.

### VTE during Asparaginase Treatment

In total, 18 of the 205 patients (8.8%; 95% confidence interval [CI]: 4.9–13.3%) developed VTE. In the induction phase, nine patients developed VTE <14 days after asparaginase. Seven out of the nine patients received one or more episodes of FFP supplementation during asparaginase administration. In all nine patients (100%) with VTE after the induction phase, thrombosis occurred <14 days after asparaginase treatment. Two patients (22%) who received FFP administration during asparaginase treatment in the induction phase also received FFP supplementation in the intensification phase. The median time from diagnosis to thrombosis was 76 days (range: 22–455 days). Thirteen patients (72%) patients developed symptomatic catheter-related thrombosis. Two patients (11%) suffered from deep vein thrombosis of the leg and three patients (17%) developed cerebral sinovenous thrombosis (CSVT). The diagnoses of VTE were confirmed with either ultrasonography ( *n* = 15), CT ( *n* = 2), or MRI ( *n* = 1).

### FFP Supplementation

In total, 82/205 patients (40%) received FFP supplementation during asparaginase treatment, 26 of 113 patients in the DCOG ALL-9 group and 56 of 92 patients in the DCOG ALL-10 group. The incidence of VTE was higher in patients with FFP (11/82) than in patients without FFP (7/123) (13 vs. 6%, *p* = 0.056).

**Fig. 3:**
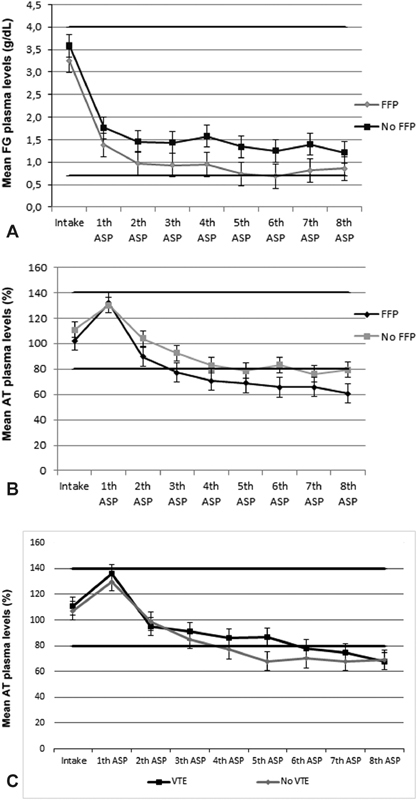
( **A** ) Mean fibrinogen (FG) plasma levels in patients during asparaginase (ASP) treatment with and without fresh frozen plasma (FFP) supplementation. ( **B** ) Mean antithrombin (AT) plasma levels in patients during ASP treatment with and without FFP supplementation. ( **C** ) Mean AT plasma levels in VTE and non-VTE patients during ASP treatment with and without FFP supplementation. VTE, venous thromboembolism.

FFP supplementation did not alter plasma levels of AT or fibrinogen ( Fig. 3 ). No differences in plasma levels of AT or fibrinogen were observed between VTE patients without and with FFP supplementation. In addition, there were no differences in fibrinogen and AT plasma levels between VTE patients and control patients after FFP supplementation.

Two patients received other therapies such as thromboprophylaxis. One patient received AT concentrate and one patient received prophylactic LMWH. None of these patients developed VTE.

### Risk Factors

In the univariate analysis, especially patients ≥12 years were at increased risk for VTE (odds ratio [OR]: 5.63; 95% CI: 2.00–16.24) ( Table 2 ). In children treated according to the ALL-10 protocol, VTE occurred significantly more often than in children treated according to the ALL-9 treatment regimen (OR: 4.89; 95% CI: 1.55–15.43). Gender, CVC, FFP supplementation, and ALL subtype were no risk factors in the univariate analysis. In the multivariate analysis, patients ≥12 years (OR: 3.89; 95% CI: 1.29–11.73) and treatment regime ALL-10 (OR: 3.71; 95% CI: 1.13–12.17) remained the only risk factors for VTE.

**Table 2:** Logistic regression analysis for risk of VTE; univariate and multivariate analyses

Variable	Univariate OR (95% CI)	*p* -Value	Multivariate OR (95% CI)	*p* -Value
Age dichotomous (≥12 versus <12 y)	5.63 (2.00–16.24)	0.001	3.89 (1.29–11.73)	0.016
Female versus male	1.82 (0.62–5.31)	0.268		
T-ALL versus B-ALL	1.82 (0.38–8.87)	0.457		
ALL-10 versus ALL-9	4.89 (1.55–15.43)	0.007	3.71 (1.13–12.17)	0.031
FFP versus no FFP	2.57 (0.95–6.93)	0.063		
CVC versus no CVC	1.10 (1.05–1.15)	0.182		

Abbreviations: ALL, acute lymphoblastic leukemia; CI, confidence interval; FFP, fresh frozen plasma; OR, odds ratio; VTE, venous thromboembolism.

### Outcome

**Fig. 4:**
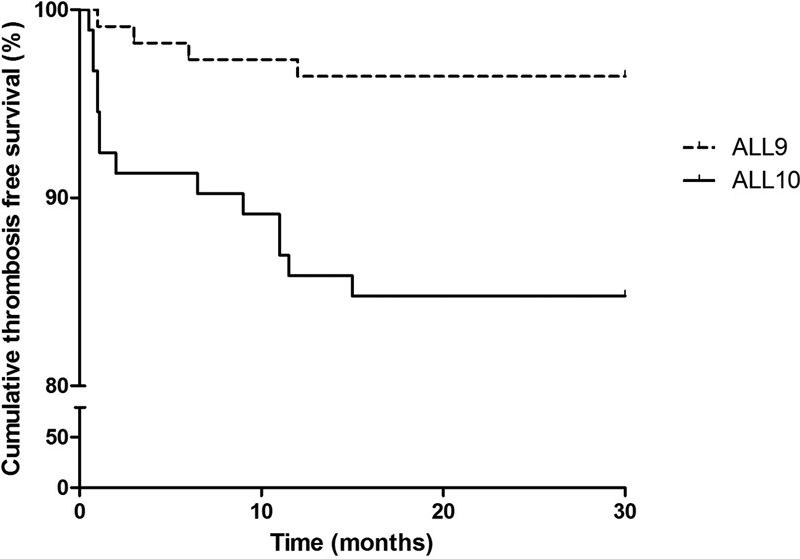
Cumulative venous thromboembolic disease-free survival of children treated according to the DCOG ALL-9 and ALL-10 treatment protocol. ALL, acute lymphoblastic leukemia; DCOG, Dutch Childhood Oncology Group.

The cumulative VTE-free survival in children treated according to the ALL-9 protocol after 1 and 2 years was both 96.5%. In children treated according to the ALL-10 protocol, the survival after 1 and 2 years was 87.0 and 84.8%, respectively (log rank test, *p* = 0.003) ( Fig. 4 ). During the whole treatment period 18 patients died. Two patients with VTE died, both as a result of ALL relapse.

## Discussion

Our study demonstrated no prophylactic effect of FFP on VTE in patients with ALL. The total symptomatic VTE incidence in DCOG ALL-9 and ALL-10 protocols was 8.8%. Age ≥12 years and treatment according to the DCOG ALL-10 protocol were independent risk factors for VTE.

We could not demonstrate a beneficial effect of FFP supplementation on the incidence of VTE during asparaginase treatment as in the adult study of Lauw et al. 12 The opposite occurred: in our study, patients who received FFP developed more VTE than patients without FFP. This result might be due to “confounding by indication.” 15 Severely decreased plasma levels of fibrinogen (and as a consequence AT) were the indication for FFP treatment, but also put patients at a higher risk for VTE.

Since the number of patients that used other antithrombotic measures besides FFP was negligible, the true antithrombotic effect of FFP was studied. The lack of effect of FFP as VTE prophylaxis in pediatric ALL patients was previously demonstrated in small pediatric studies. 16 17 18 19 However, in adult ALL patients, a 70% decrease in incidence of VTE occurred after prophylactic FFP supplementation, despite the absence of rise in AT and fibrinogen levels in these patients. 12 Also in our study, no increase in fibrinogen and AT levels after FFP supplementation was observed. FFP infusion of 1 mL/kg is expected to give a rise of 1.6% of AT. 20 As a consequence, the low advised pediatric dosage of 10 mL/kg in the DCOG protocols might be an explanation for the absence of effects.

The incidence of symptomatic VTE in our study was in concordance with previous studies in ALL patients. 1 2 VTE seemed to occur more often in patients treated according to DCOG ALL-10 than in children treated according to the DCOG ALL-9 protocol. Moreover, the cumulative VTE-free survival was significantly lower in DCOG ALL-10 patients. In the induction phase of DCOG ALL-10, patients received approximately twice as much asparaginase within a longer duration, in combination with prednisone, compared with the DCOG ALL-9 protocol. Asparaginase induces a hypercoagulable state. 21 Longer duration of asparaginase is an extra risk factor. 21 Furthermore, patients treated within the DCOG ALL-10 protocol received prednisone, while patients in DCOG ALL-9 got dexamethasone. A large multicenter study in pediatric ALL patients showed a significant reduction of VTE with the use of dexamethasone instead of prednisone. 22 Corticosteroids, especially prednisone, creates a hypercoagulable and hypofibrinolytic state, amplifying the hypercoagulable effect of asparaginase. 23 24 25 Finally, in contrast to the ALL-9 protocol, asparaginase was continued after the induction phase, in the intensification phase of the MR patients, causing another nine patients to develop VTE. Additionally, age was an independent risk factor. VTE developed significantly more frequently in patients of 12 to 18 years old than in children of 1 to 11 years old. This was shown in previous studies, as well. In a national ALL study in the Netherlands, an age ≥7 years increased the risk of VTE, and in another study there was an increased risk of VTE in children aged ≥10 years. 26 27 Older age as a risk factor can be partly explained by alterations in the hemostatic system. The capacity of thrombin generation during infancy and childhood is lower compared with adults. The capacity increases with age and reaches adult values during adolescence. 28 29

CVC-related VTE was the most common presentation of VTE. The relationship between CVC and VTE in ALL patients was well described in previous studies. 4 30 In 25% of the cases, VTE occurred as a CSVT, a potentially severe complication, with a high case-fatality rate of 0 to 29%. 31 32 33 The incidence of CSVT in ALL patients is considerably higher compared with the normal pediatric population. 33 Previous studies have shown that patients during ALL treatment are at an increased risk for CSVT. 32 34 The pathophysiology is still unclear. 32 33 35 As in the study by Abbott et al, the current study did not seem to show beneficial effect of FFP for the prevention of CSVT. 31

Besides FFP, several other interventions have been studied, such as thromboprophylaxis in ALL patients. AT supplementation was investigated in the PARKAA Study. Although this study was not powered to establish a significant effect, a trend was seen in VTE reduction after supplementation of AT. 36 Very recently, the Thrombotect trial showed a lower risk of VTE in children with prophylactic AT concentrate (1.9%) than in children with low-dose unfractionated heparin (8%). 37 However, the 5-year event-free survival was decreased in the AT group (80.9 ± 2%) compared with the unfractionated heparin group (85.9 ± 2%). Mitchell et al showed that prophylactic LMWH effectively reduced the risk of VTE in patients at high risk for VTE. 38 Also, other pilot studies with prophylactic LMWH showed encouraging results. 39 40 In the Thrombotect trial the VTE risk with prophylactic LMWH was 3.5% with a 5-year event-free survival of 86.2 ± 2%. 37 A drawback of this therapy is the subcutaneous administration. In the past decade, the DOACs have been introduced. 41 42 43 44 45 Currently, apixaban is being studied in pediatric ALL patients as a tromboprophylaxis agent. 46 47

This study has its limitations. As mentioned above, our most important limitation is the confounding bias. Furthermore, its retrospective design and unequal distribution of patients aged ≥12 and presence of a CVC between ALL-9 and ALL-10 treatment protocols may have caused a selection bias. However, all patients were subsequently recorded in the EKZ/AMC oncology database and all medical data were reviewed precisely. Furthermore, after including presence of CVC in the multivariate analysis, protocol type and age remained independent risk factors (data not shown). In addition, due to its retrospective design, randomization for FFP did not occur. Nevertheless, this study represents a large cohort of ALL patients treated according to two standardized protocols. Moreover, the aims of this study did not include survival of patients with and without FFP, so it was impossible to study the effect of FFP on survival. Finally, VTE occurred only in a small number of patients, which might have influenced the analysis of the association between VTE and risk factors.

In conclusion, FFP supplementation does not seem to be beneficial in the prevention of VTE in pediatric ALL patients. In addition, age ≥12 years and the DCOG ALL-10 protocol with intensive and prolonged treatment with asparaginase in combination with prednisone appeared to be VTE risk factors in the current study. There is a need for effective preventive strategies in ALL patients at high risk for VTE.
